# *Pseudomonas aeruginosa* RRALC3 Enhances the Biomass, Nutrient and Carbon Contents of *Pongamia pinnata* Seedlings in Degraded Forest Soil

**DOI:** 10.1371/journal.pone.0139881

**Published:** 2015-10-13

**Authors:** Parthasarathy Radhapriya, Andimuthu Ramachandran, Rangasamy Anandham, Sundararajan Mahalingam

**Affiliations:** 1 Centre for Climate Change and Adaptation Research, Anna University, Chennai, 600025 Tamil Nadu, India; 2 Department of Agricultural Microbiology, Agricultural College and Research Institute, Tamil Nadu Agricultural University, Madurai 625104, Tamil Nadu, India; 3 Centre for Biotechnology, Anna University, Chennai, 600025 Tamil Nadu, India; Graz University of Technology (TU Graz), AUSTRIA

## Abstract

The study was aimed at assessing the effects of indigenous Plant Growth Promoting Bacterium (PGPB) on the legume *Pongamia pinnata* in the degraded soil of the Nanmangalam Reserve Forest (NRF) under nursery conditions. In total, 160 diazotrophs were isolated from three different nitrogen-free semi-solid media (LGI, Nfb, and JMV). Amongst these isolates, *Pseudomonas aeruginosa* RRALC3 exhibited the maximum ammonia production and hence was selected for further studies. RRALC3 was found to possess multiple plant growth promoting traits such as nitrogen accumulation (120.6ppm); it yielded a positive amplicon with *nifH* specific primers, tested positive for Indole Acetic Acid (IAA; 18.3μg/ml) and siderophore production, tested negative for HCN production and was observed to promote solubilization of phosphate, silicate and zinc in the plate assay. The 16S rDNA sequence of RRALC3 exhibited 99% sequence similarity to *Pseudomonas aeruginosa* JCM5962. Absence of virulence genes and non-hemolytic activity indicated that RRALC3 is unlikely to be a human pathogen. When the effects of RRALC3 on promotion of plant growth was tested in *Pongamia pinnata*, it was observed that in *Pongamia* seedlings treated with a combination of RRALC3 and chemical fertilizer, the dry matter increased by 30.75%. Nitrogen, phosphorus and potassium uptake increased by 34.1%, 27.08%, and 31.84%, respectively, when compared to control. Significant enhancement of total sugar, amino acids and organic acids content, by 23.4%, 29.39%, and 26.53% respectively, was seen in the root exudates of *P*. *pinnata*. The carbon content appreciated by 4-fold, when fertilized seedlings were treated with RRALC3. From the logistic equation, the rapid C accumulation time of *Pongamia* was computed as 43 days longer than the control when a combination of native PGPB and inorganic fertilizer was applied. The rapid accumulation time of N, P and K in *Pongamia* when treated with the same combination as above was 15, 40 and 33 days longer, respectively, as compared to the control.

## Introduction

Soil degradation is a serious worldwide environmental problem that leads to poor soil health [1]. Soil degradation involves an alteration process that negatively impacts the characteristics of a forest, in particular, by reducing the value of its goods and services, and may vary in frequency, quality, extent, origin, and severity. It can be caused by various disturbances: natural or anthropogenic or a combination thereof [2]. Enhancing carbon sequestration in degraded forests is an important task in tropical countries. Reversing forest degradation through restoration has been observed to increase carbon stocks. However, natural regeneration and replantation are difficult tasks in tropical degraded forests [3] as soils have already lost their nutrient content, this adversely affects plant growth as well as the regeneration of new saplings [4]. Although the use of chemical fertilizers and manures in forests and forest nursery production is insignificant when compared with the agricultural sector, adoption of this practice could lead to nutrient deficiency and severe environmental damage in degraded forests [5]. Moreover, the use of such additives to enhance the soil nutrient status and crop yield can lead to pollution of forest soil and put the complex system of biogeochemical cycles under pressure [6]. Environmental degradation occurs when the leachate of added nutrients, like nitrogen and phosphorus, mixes with the runoff [7, 8]. Therefore, there is an increasing interest in the development of eco-friendly plant growth promoting bacterium (PGPB), which is known to improve growth in agricultural crops, to help enhance plant growth in an ecologically sustainable manner in degraded forests [9, 10].

Several studies have confirmed the positive effect of PGPB on the yields of different agricultural crops in varying climatic conditions and soils [11–15]. PGPB stimulates plant growth by direct as well as indirect mechanisms such as nitrogen fixation, mineral solubilization, phytohormone production and antagonistic activities [16]. It is hypothesized that PGPB may facilitate improvement in the soil health and productivity of forests and forest nursery plants. However, PGPB have not yet been studied in afforestation processes like repopulation of native plants, restoration after forest fire, prevention of soil erosion, soil reclamation in mining regions and reforestation [17]. Bashan *et al*. [18] reported that the inoculation of *Prosopis articulata*, *Parkinsonia microphylla*, *Parkinsonia florida* with native PGPB enhanced the development of plant shoot and root, increased the number of branches and improved the survival rate in forest seedlings. PGPB inoculation along with fertilizer has been reported to increase dry matter accumulation and nutrient uptake in the *Fraxinus americana* forest seedlings [19].

The study area, Nanmangalam Reserve Forest (NRF), is located in the southern part of Chennai, Tamil Nadu, India. It covers an area of 321ha, with latitudinal extension from 12°55’5” N to 12°56’13” N and longitudinal extension from 80°9’46” E to 80°10’57” E. NRF is a degraded reserve forest and its soil is reported to be deficient in nutrients such as total nitrogen, organic carbon, available potassium, calcium, magnesium, iron, and zinc [20]. The trees exhibit stunted growth with a maximum height of 2 m and the crown cover is less than 30% with very low biomass. The tree population was found to be highly scattered and no regeneration of tree species was observed. The soil of this locality seemed to be completely eroded, devoid of significant topsoil and most of the forest was exposed to the weathering of rocks.

To the best of our knowledge, the potential of native PGPB to enhance the biomass of forest seedlings has not yet been studied in India. Therefore, in the present study, a novel attempt has been made to isolate an indigenous bacterium from a degraded forest and to understand its influence on the growth, biomass and nutrient content of *Pongamia pinnata*.

## Materials and Methods

### Isolation of PGPB from NRF

Since NRF is a reserve forest, official permission to conduct experiments was acquired from the Principle Chief Conservator of Forest, Department of Forest, Tamil Nadu, India. We confirm that the field studies carried out did not involve any endangered or protected species.

For systematic soil sampling, the entire area was divided into 40 grids of 0.25 km^2^ each. In total, 40 samples were collected. To reduce the number of soil samples, four samples each were pooled and homogenized by thorough mixing. Thus, a total of ten samples were used for the isolation of efficient PGPB. The samples were placed in sterile bags, immediately transported to the laboratory and processed.

The soil samples were inoculated into 5ml each of three different nitrogen-free semisolid media (Johanna Mannitol Vera-JMV; Loitsyanskya, Gv and Ivchenko-LGI and New Fábio Pedrosa-Nfb). In the LGI medium, sucrose was used as the carbon source and pH was adjusted to 6.0–6.2. In JMV and Nfb media, mannitol and malic acid were used as carbon sources and the pH was adjusted to 6.0–6.2 and 6.0–6.5, respectively. The vials were incubated at 30°C and the formation of a white subsurface pellicle was observed at the end of 5 days [21]. The vial with the highest dilution that was still showing pellicle formation was transferred to new sterile semisolid medium for further confirmation. Five days after pellicle formation, the cells were transferred to nitrogen-free solid medium Petri dishes for isolation of single colonies. The isolated colonies were preserved in 50% glycerol at −80°C for further study.

Different plant growth promoting characteristics were examined in the isolated bacteria. Ammonia production was estimated by the colorimetric method proposed by Cappuccino and Sherman [22]. Amongst the tested isolates, RRALC3 exhibited maximum ammonia production and was therefore selected for further study. The nitrogen content was analyzed by the micro-Kjeldahl method [23]. The presence of *nifH* gene was detected by PCR using the 16F (5′-GCIWTYTAYGGIAARGGIGG-3′) and 407R (5′-AAICCRCCRCAIACIACRTC-3′) gene specific primers [24]. Indole acetic acid (IAA) production was determined as per the methodology proposed by Gorden and Webber [25]. Qualitative determination of hydrocyanic acid (HCN) production was performed as per the method recommended by Kremer and Souissi [26]. Phosphate solubilization was demonstrated on Pikovskaya’s agar plates as described by Gaur [27]. Zinc and silicate solubilization were examined in Bunt and Rovira medium [28] supplemented with 0.1% zinc oxide and KAl Si_3_O_8_, respectively. A qualitative assay for siderophore production was conducted on CAS agar plates [29].

### Molecular characterization

Bacterial isolate RRALC3 was grown in LGI medium. DNA was extracted according to Sambrook *et al*. [30]. The bacterial 16S rDNA gene was amplified by PCR using the forward primer 27F (5′-AGAGTTTGATCCTGGCTCAG-3′) and reverse primer 1492R (5′-GGTTACCTTGTTACGACTT-3′). The 16S rDNA nucleotide sequence was determined by direct PCR sequencing using the fluorescent dye terminator method (ABI Prism^TM^ Bigdye^TM^ Terminator cycle sequencing ready reaction kit v.3.1). The products were purified with a Millipore-Montage dye removal kit and run in an ABI3730XL capillary DNA sequencer (50 cm capillary). The nearly complete 16S rDNA gene sequences obtained from the automatic sequencer was aligned and the EzTaxon-e server was used to determine bacterial identity as well as to ascertain closest relatives [31]. The bacterial isolate RRALC3 obtained in this study exhibited 99% 16S rDNA gene sequence similarity to *Pseudomonas aeruginosa* JCM5962. The sequence was submitted to the NCBI database (accession number KF481965).

### Detection of pathogenicity

#### Hemolytic activity

To assay for the production of haemolysin, exotoxins capable of destroying Red Blood Cells (RBC) and hemoglobin, three strains of *Pseudomonas*: *P*. *aeruginosa* RRALC3, *P*. *aeruginosa* PA14 (ATCC 15442) and *P*. *aeruginosa* PA01(ATCC 15692) were plated on sheep blood agar plates (catalogue no. MP1301, Himedia, India). The plates were incubated for 48–72 h and scrutinized for the appearance of clear, greenish-brown and no zone which is indicative of complete (β), partial (α) and no (ɤ) hemolytic activity, respectively.

#### Detection of pathogenic islands

In order to confirm the existence of pathogenic islands, RRALC3 was examined for the presence of virulence genes. PCR amplification of the *ecfX* (411 bps), *ybtQ* (387 bps) and *lasB* (731 bps) genes was performed in 25 μl reaction mixtures containing 2.5μl of dNTPs (10mM), 1.25 μl each of forward and reverse primers (5 pmol each), 2.5 μl 10x PCR buffer and 0.25 μl Taq DNA polymerase (3 μl; Genei, India). The following gene-specific primers were used: (a) *ecfX* F (TCCGTGGTTCCGTCTCGCATG) and *ecfX* R (CGACCTGGGACAGGCCGTCGAA) [32], (b) *ybtQ* F (CGAGGCCCTACTCAGGACGCCTCA) and *ybtQ* R (AGGGCCAAGGGACCGACTGCCA) [33], and (c) *lasB* F (CGAGCCAGGGGAGTGCAGTTC) and *lasB* R (GGCCGCCCGCCTCGGCGTGGGCC) [34]. *P*. *aeruginosa* PA14 and *E*. *coli* DH5 alpha strains were used as positive and negative controls, respectively. The PCR conditions employed were as follows: initial denaturation at 95°C for 7 min, 30 cycles of denaturation at 95°C for 1 min, annealing at 51°C, 54°C or 55°C for 1min (for *ecfX*, *ybtQ* and *lasB*, respectively), and extension at 72°C for 30 s, followed by a final extension at 72°C for 10 min.

#### Phylogenetic analysis

The 16S rRNA gene sequences of *P*. *aeruginosa* RRALC3 was aligned with clinical and environmental *P*. *aeruginosa* isolates retrieved from the NCBI database using CLUSTAL W (version 1.8). A phylogenetic dendrogram was constructed by the neighbor-joining method [35].

### Effect of inoculation of *P*. *aeruginosa* on *P*. *pinnata*

#### Preparation of bacterial inoculum and fertilizer

In order to prepare *P*. *aeruginosa* inoculum the bacterium was grown in LGI medium at 37°C for 96 h. Cells were harvested by centrifugation at 5,000 ×*g*, 4°C for 10 min. The cell pellet was washed twice and resuspended in 0.1 M phosphate buffer in order to obtain a cell density of 10^6^ cfu/ml. A commercial biofertilizer (National Fertilizer Ltd., Noida, India) (10 kg) containing 10^5^ CFU each of *Azospirillum*, *Azotobacter* and *Rhizobium* per gram of dry lignite powder was mixed with 50 kg of sand and spread over 1 acre of land. Inorganic fertilizer (1.5 g), comprising of N, P, and K at the ratio of 2:1:1, was added per cubic meter of soil.

#### Mother bed preparation

Five mother beds, of size 12.5 × 1.2 m each, were prepared by digging and hoeing. Five-hundred *Pongamia* seeds (Tambaram Nursery, Tamil Nadu Forest Department, Chennai) were surface sterilized by treating with 0.1% HgCl_2_ for 2 min followed by rinsing six times with sterile distilled water. Surface sterilization was performed to eliminate surface microorganisms and seed borne pathogens. The seeds were sown in the mother bed and subjected to the following treatments:

T1: Inorganic fertilizer (N:P:K, 2:1:1) + *P*. *aeruginosa* RRALC3
T2: Inorganic fertilizer (N:P:K, 2:1:1) + commercial biofertilizer
T3: Inorganic fertilizer (N:P:K, 2:1:1)
T4: *P*. *aeruginosa* RRALC3
T5: No biofertilizer and no inorganic fertilizer (control)

Thirty days old seedlings were transferred from the mother bed to a polyethylene bag (30×45 cm) containing 10 kg of soil prepared in the same fashion as described for the mother bed. The experiment was conducted in completely randomized block design (CRD) and each of the five treatments was replicated three times. A total of 450 healthy seedlings, 90 seedlings per treatment, were used for the nursery treatments. The physico-chemical properties of the soil used for experiments were as follows: Soil texture, clayey; bulk density, 1.02; pH, 6.8; soil organic carbon (SOC), 1.10%; nitrogen (N), 0.19%; available phosphorus, 1.66 mg kg^−1^; available potassium, 1.51 (cmol kg^–1^); calcium (Ca), 7.6 mg kg^–1^; magnesium (Mg), 3.92 mg kg^–1^; iron (Fe), 0.31 mg kg^–1^; manganese (Mn), 0.28mg kg^–1^; copper (Cu), 0.28mg kg^–1^ and zinc (Zn), 0.16mg kg^–1^.

### Data collection for plant analysis

The experiment was conducted over a period of 180 days (October 2012 to March 2013). To set up the logistic equation, dry matter accumulation (DMA) and nutrient content of the above ground biomass of the plant samples were analyzed at 30 days intervals. Three replicate samples were harvested. The polythene bags were flooded with water to loosen soil from the plant roots and seedlings were uprooted without any injury. The roots, stems, and leaves of the seedlings were then separately harvested and washed thoroughly with distilled water to remove the soil and other debris. The plants were then placed in paper bags and kept at 70°C to attain a constant weight. The dried plant material was crushed and passed through a 250 μm mesh sieve prior to determining the carbon (C), nitrogen (N), phosphorus (P), potassium (K), magnesium (Mg), calcium (Ca), and sulphur (S) contents. C, N and S contents were quantified using a CHNS-O elemental analyzer (Vario EL cube, Germany) as described previously by Wang and Anderson [36]. The total P content was determined by the ascorbic acid method [37]. K, Ca and Mg contents were determined spectrophotometrically using a Perkin Elmer Lambda 25 UV/Vis spectrophotometer as described by Johnson and Ulrich [38].

To examine the components of the root exudates, replicated samples of the rhizosphere soil were collected [19]. Within 12 h of sampling, soil solution was removed using double-bottomed centrifuge cups and immediately filtered through Corning disposable syringe filters (0.22 μm filter). The soil solution was prepared using soil and distilled water in the ratio of 1:3.3 to obtain a good equilibrium. The centrifugation was carried out at 8000 rpm at 4°C for 30 min. The resultant supernatant was considered as the root exudates. The total protein in exudates was analyzed by the Bradford method using bovine serum albumin as standard [39]. Carbohydrate concentration of root exudates was determined using the heptafluorobutyrate derivatives of O-ethyl-glycosides obtained after methanolysis in 0.5 M methanol—HCl for 24 h at 80°C. The samples were analyzed by gas chromatography using a capillary column (25×0.32 mm) of 5% silicone OV 210 in a Varian 2100 gas chromatograph (France) equipped with a flame detector and a glass solid injector. The carrier gas was helium and the oven temperature was programmed to increase from 150°C to 250°C in 3 min. Lysine, which can be derivatized as a carbohydrate heptafluorobutyrate and maintain a different retention time in gas chromatography as compared to the sample gas, was used as an internal standard. For amino acid analysis, a 100 μg sample was hydrolyzed under vacuum with 2 ml of 6M HCl at 110°C in sealed tubes for 48 h. A drop of phenol was added to avoid degradation of tyrosine residues. The samples were analyzed in an automatic amino acid analyzer (Durrum 500, Dionex-Durram Chemical Corporation, London, UK) using norleucine as an internal standard [40].

### Soil analysis

Soil samples were tested at the end of the experiment. A pH meter was used to measure pH of 2:1 water:soil suspension. Soil organic C, N and S contents were determined using a CHNS-O elemental analyzer (Vario EL cube) as described previously [35]. Micro-nutrient content of the soil was analyzed by DTPA extraction methods as outlined by Lindsey and Norwell [41]. The available P and exchangeable cations were analyzed according to Olsen and Sommers [42].

### Statistical analysis

The effect of the different treatments on dry matter accumulation (DMA) of *P*. *pinnata* was examined using the analysis of variance (ANOVA) and the least significant difference at 5%. These tests were used to compare the means of treatments using the SAS software (SAS version 9.2 windows for 32- licensed version).

Nutrient accumulation in the above ground biomass of *P*. *pinnata* was described using the logistic equation *W* = *a*/[1 + exp ^(b − ct)^] [43], where *W* is the nutrient accumulation variable at days after emergence *t*, and *a*, *b*, *c* are values calculated using the SAS software. Plant growth, growth rates, and growth acceleration curves were obtained using the first, second and third derivatives of this logistic equation. *V*_m_, *T*_m_, *T*_*a*_, and *T*_*b*_ were determined by calculation based on *a*, *b* and *c* nutrient accumulation of the logistic equation (*V*_m_ and *T*_m_, maximum daily nutrient accumulation in mg per plant; *T*_*a*_ and *T*_*b*_, onset and termination time of rapid nutrient accumulation in days). ANOVA test was carried out to evaluate the total plant nutrient accumulation after 6 months.

## Results

### PGPB characteristics

In total, 160 diazotrophs were obtained from the three different nitrogen-free semisolid media viz., 56 from LGI, 57 from JMV and 47 from Nfb (S1 Fig). All the isolates were tested for ammonia production. Isolate RRALC3 showed maximum amount of ammonia production (8%). Further, this isolate was tested for the presence of multiple plant growth promoting traits. RRALC3 accumulated nitrogen (120.6 ±1.52 ppm) and produced IAA (18.3 ±0.12 μg ml^−1^) in the liquid medium under test conditions. It was able to grow in nitrogen-free semisolid LGI medium and yielded a positive PCR amplicon of 390 bp with *nif H* specific primers (S2 Fig). The isolated bacterium RRALC3 was able to solubilize P, Si and Zn in the plate assay as evidenced by the formation of a clear halo zone around the bacterial colony. CAS plates exhibited an orange zone which is indicative of siderophore production. RRALC3 did not produce HCN under the tested conditions.

### Detection of pathogenicity in *Pseudomonas aeruginosa* RRALC3

**Fig 1:**
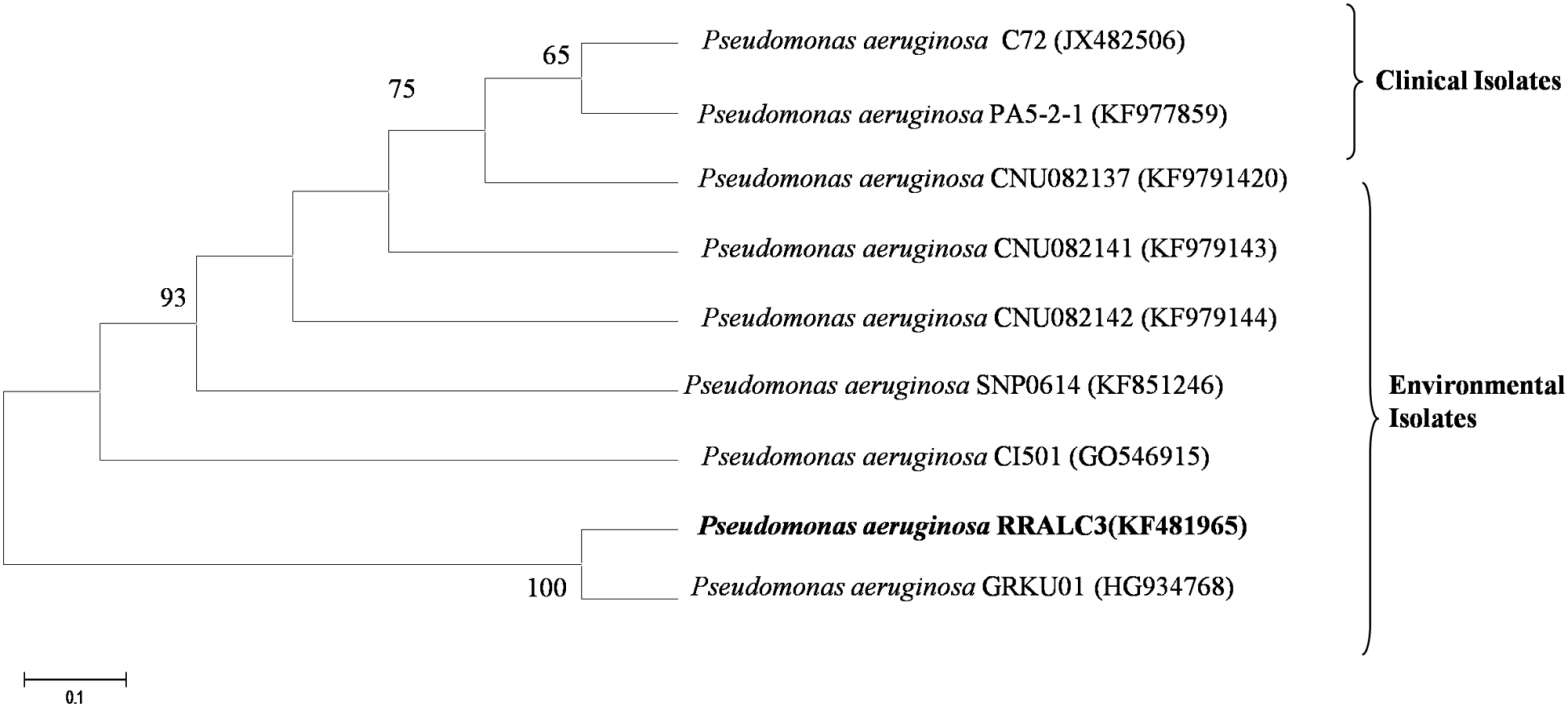
Phylogenetic tree based on 16S rRNA gene sequences. Bar, 0.1 nucleotide changes per positions. *Pseudomonas aeruginosa* RRALC3 obtained in this study are shown in bold. Bootstrap value ≥ 50 based on 1000 resamplings are shown at the branch points.

*Pseudomonas aeruginosa* RRALC3, PA14 and PA01 were grown in blood agar medium. Isolates RRALC3 and PA01 did not exhibit any hemolytic activity whereas isolate PA14 was positive for α-hemolysis (S3 Fig). Virulence genes responsible for pathogenesis were examined in *P*. *aeruginosa*. RRALC3 was examined for the presence of pathogenesis related virulence genes using a PCR assay with degenerative primers. The results clearly indicated that virulence genes *ecfX*, *ybtQ* and *lasB* were absent in RRALC3 (S4 Fig). A neighbor joining phylogenetic tree was constructed with 16S rDNA sequences of clinical and environmental *P*. *aeruginosa* isolates. The environmental and clinical isolates were clustered separately which was supported by high bootstrap values (Fig 1).

### Dry matter accumulation in different treatments

**Fig 2:**
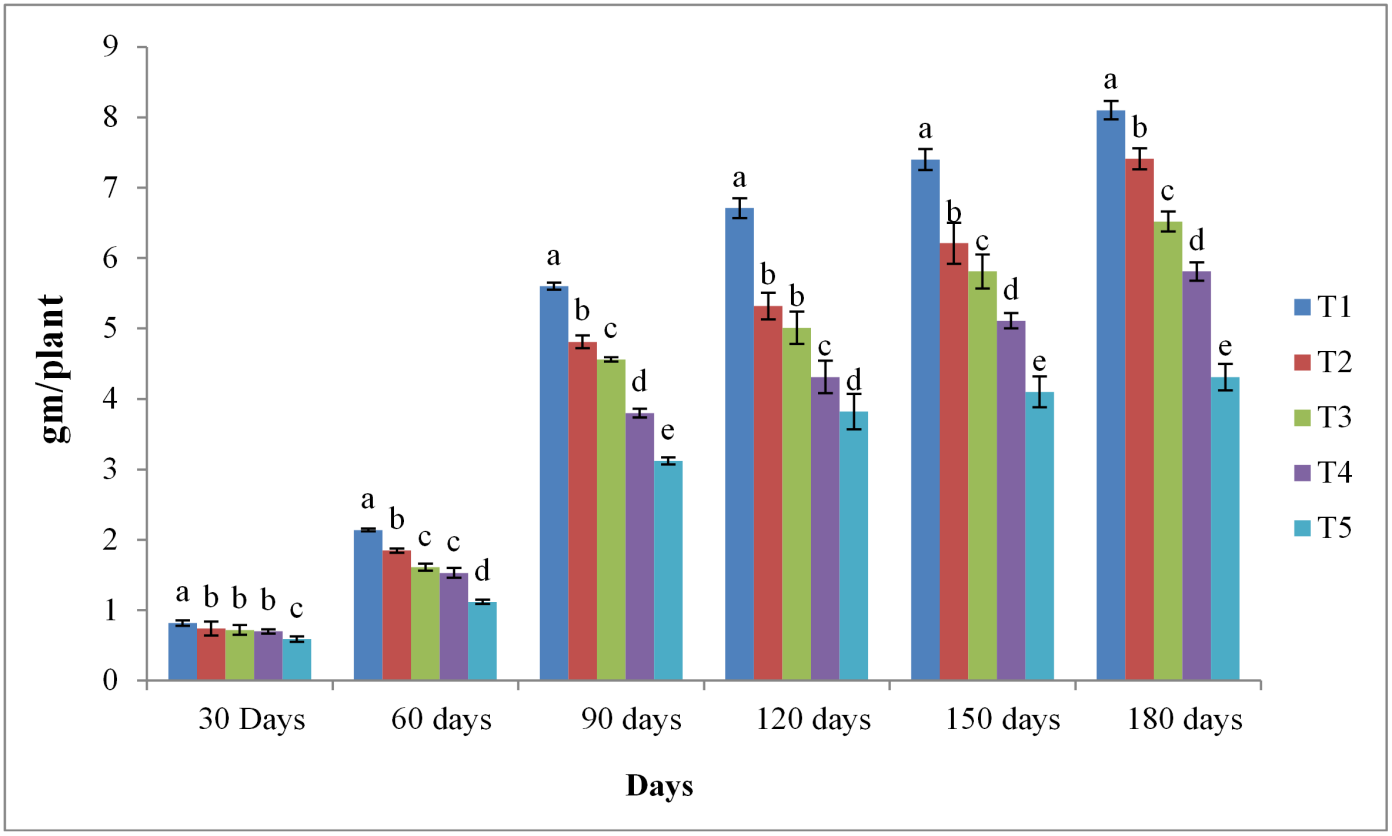
Effects of different treatments on the dry matter accumulation of *Pongamia pinnata* seedlings. ^1^ DMA, dry matter accumulation. Values represent the mean ± SD of three replicates. ^2^ Values mentioned on the bar-chart are significantly different (*P* < 0.05) among treatments. ^3^ T1, inorganic fertilizer (N:P:K, 2:1:1) + *P*. *aeruginosa* RRALC3; T2, inorganic fertilizer (N:P:K, 2:1:1) + commercial biofertilizer; T3, inorganic fertilizer (N:P:K, 2:1:1); T4, *P*. *aeruginosa* RRALC3; T5, control (no biofertilizer or inorganic fertilizer).

On day 30, the Dry Matter Accumulation in the aerial parts of *P*. *pinnata* showed a significant difference between T1 (inorganic fertilizer with *P*. *aeruginosa* RRALC3) and T5 (control) treatments (Fig 2). On day 60, no significant difference was observed between T2 (inorganic fertilizer with commercial biofertilizer) and T3 (inorganic fertilizer alone) treatments; however, a highly significant difference was observed between T1 and T4 (*P*. *aeruginosa* alone) treatments. On day 180, a maximum of 25.8% DMA was observed in T1. At the same time, a small difference was observed between T2 (21.7%) and T3 treated (20.5%) seedlings. However, during the entire experimental period, T5 showed no significant impact on DMA as compared to the other treatments (Fig 2).

### Nutrient content and plant biomass

Macro nutrients like N, P and K and micro nutrients like Mg, S and Ca were increased by 12.4%, 12.1%, and 8.9%, and 2.4%, 2%, and 3%, respectively in T1 treatment (Table 1). The difference in N, P and K between T1 and T2 treated plants was 13%, 2%, and 6% respectively. The maximum growth in the above ground biomass was observed predominantly in shoots of the treated plants across all treatments. The above and below ground biomass increase in different treatments was in the following order: chemically fertilized seedlings with *P*. *aeruginosa* (T1) > commercial fertilizer with inorganic fertilizer (T2) > inorganic fertilizer (T3) > PGPB alone (T4) > control (T5) (33.7% > 28.3% > 17.3% > 12.19% > 8.5%, respectively). The C content varied significantly under different treatments and the variance was in the following order: T1 > T2 > T4 > T3 > T5 (37.43% > 21.7% > 18.13% > 15.7% > 7.8%, respectively) (Table 1).

**Table 1:** Total uptake of different nutrients and carbon content in *Pongamia pinnata* seedlings after180 days of growth.

Nutrient Content	Shoot		Root^1^
	Stem^1^	Leaves^1^	
**N(%)**			
**T1**	1.961±0.2^a^	1.062±0.08^a^	1.321±0.07^a^
**T2**	0.934±0.01^b^	0.626±0.01^b^	0.562±0.02^b^
**T3**	0.456±0.01^c^	0.419±0.01^c^	0.323±0.01^c^
**T4**	0.417±0.01^d^	0.394±0.02^d^	0.305±0.01^d^
**T5**	0.403±0.01^d^	0.397±0.01^d^	0.304±0.01^d^
**P (%)**			
**T1**	2.122±0.3^a^	2.121±0.02^a^	2.462±0.27^a^
**T2**	1.436±0.15^b^	1.050±0.23^b^	1.120±0.08^b^
**T3**	0.841±0.02^c^	1.111±0.12^b^	0.975±0.02^c^
**T4**	0.731±0.01^d^	0.569±0.11^c^	0.726±0.03^d^
**T5**	0.723±0.01^d^	0.418±0.02^c^	0.412±0.04^e^
**K(%)**			
**T1**	1.311±0.5^a^	1.369±0.6^a^	1.296±0.2^a^
**T2**	0.961±0.3^b^	1.010±0.02^b^	0.951±0.03^b^
**T3**	0.911±0.2^c^	0.841±0.01^c^	0.847±0.01^c^
**T4**	0.414±0.01^d^	0.412±0.02^d^	0.362±0.02^d^
**T5**	0.395±0.01^d^	0.410±0.02^d^	0.248±0.01^e^
**Mg(%)**			
**T1**	0.101±0.001^a^	0.093±0.001^a^	0.089±0.001^a^
**T2**	0.098±0.001^b^	0.079±0.001^b^	0.081±0.002^a^
**T3**	0.029±0.001^d^	0.029±0.001^d^	0.024±0.001^c^
**T4**	0.056±0.002^c^	0.065±0.001^c^	0.049±0.002^b^
**T5**	0.024±0.002^d^	0.021±0.001^d^	0.021±0.001^c^
**S(%)**			
**T1**	0.095±0.001^a^	0.022±0.001^e^	0.076±0.001^a^
**T2**	0.077±0.001^b^	0.055±0.001^b^	0.054±0.002^b^
**T3**	0.038±0.001^d^	0.031±0.001^d^	0.042±0.003^c^
**T4**	0.060±0.001^c^	0.042±0.001^c^	0.051±0.001^d^
**T5**	0.027±0.001^e^	0.072±0.001^a^	0.024±0.001^e^
**Ca(%)**			
**T1**	0.310±0.001^a^	0.226±0.001^a^	0.205±0.001^a^
**T2**	0.269±0.001^b^	0.201±0.002^b^	0.201±0.002^b^
**T3**	0.170±0.001^d^	0.151±0.001^d^	0.111±0.001^d^
**T4**	0.257±0.001^c^	0.196±0.001^c^	0.176±0.001^c^
**T5**	0.167±0.001^d^	0.110±0.002^e^	0.099±0.001^de^
**C (%)**			
**T1**	33.911±1.4^a^	19.911±0.84^a^	21.120±1.01^a^
**T2**	19.063±1.08^b^	8.310±0.59^b^	16.162±0.78^b^
**T3**	14.345±0.87^d^	6.028±0.38^c,d^	11.229±0.57^d^
**T4**	16.975±0.78^c^	7.544±0.41^c^	12.438±0.6^c^
**T5**	7.492±0.7^e^	2.949±0.2^e^	5.455±0.41^e^

^1^ Values followed by different uppercase letters in a column are significantly different (*P* < 0.05) among treatments.

T1, Inorganic fertilizer (N: P: K– 2:1:1) + *P*. *aeruginosa* RRALC3; T2, Inorganic fertilizer (N: P: K– 2:1:1) + commercial biofertilizer; T3, Inorganic fertilizer (N: P: K– 2:1:1); T4, *P*. *aeruginosa* RRALC3; T5, control (no biofertilizer and inorganic fertilizer)

### Analysis of root exudates components of *P*. *pinnata*

Analysis of the root exudates revealed that the T1 treatment significantly enhanced the total sugar, amino acids and organic acids components, 23.4%, 29.39%, and 26.53%, respectively when compared with the un-inoculated control (T5) (Table 2).

**Table 2:** Root exudates componanents at 180 days after the emergence of *Pongamia pinnata* seedlings.

Treatment	Total sugar (mg kg^−1^ of root exudates)^1^	Amino acids (mg kg^−1^ of root exudates)^1^	Organic acids (mg kg^−1^ of root exudates)^1^
**T1**	291.45±31.37^a^	14.67±3.5^a^	0.082±0.021^a^
**T2**	271.92±45.98^b^	11.21±2.53^b^	0.071±0.012^b^
**T3**	231.32±56.79^d^	9.15±1.91^d^	0.065±0.031^d^
**T4**	254.67±51.74^c^	9.99±2.1^c^	0.059±0.018^c^
**T5**	191.45±27.12^d^	4.89±3.2^e^	0.032±0.019^e^

^1^ Values followed by different uppercase letters in a column are significantly different (*P* < 0.05) with in treatments.

T1, inorganic fertilizer (N:P:K, 2:1:1) + *P*. *aeruginosa* RRALC3; T2, inorganic fertilizer (N:P:K, 2:1:1) + commercial biofertilizer; T3, inorganic fertilizer (N:P:K, 2:1:1); T4, *P*. *aeruginosa* RRALC3; T5, control (no biofertilizer or inorganic fertilizer)

### Soil macro- and micro-nutrients

The addition of PGPB and inorganic fertilizers significantly altered the soil macro and micronutrients (Table 3). At the beginning of the treatment, the soil organic C content was 1.10%, but after the treatment it increased to 1.6% in the T1 treatment and 1.41% in the T2 treatment. An increase in available P content was observed in T1, T2 and T3 treated soil as well (4.91, 3.65 and 4.01 mg kg^−1^). Soil K, Ca, Mg, Fe, Mn and Zn contents were also significantly increased in T1.

**Table 3:** Effects of the treatments on macro- and micronutrients in the experimental soil.

Parameter	After Treatment (180 days)				
	T1^1^ ^,^ ^2^	T2^1^ ^,^ ^2^	T3^1^ ^,^ ^2^	T4^1^ ^,^ ^2^	T5^1^ ^,^ ^2^
**Ph**	5.4±1.3^e^	5.8±1.3^d^	6.3±1.7^b^	5.8±1.5^d^	6.51±0.5^b^
**Soil Organic Carbon (%)**	1.6±0.2^a^	1.41±0.3^b^	1.23±0.21^d^	1.38±0.7^c^	1.19±0.5^e^
**Total N (%)**	0.18±0.01^a^	0.16±0.08^b^	0.13±0.02^c^	0.14±0.04^c^	0.14±0.09^c^
**Available P (mg kg** ^**-1**^ **)**	4.91±1.7^a^	4.01±1.1^b^	3.31±0.8^c^	3.65±1.4^c^	1.59±0.85^d^
**K (cmol kg** ^**-1**^ **)**	1.37±0.94^d^	1.31±0.75^d^	1.26±0.89^c^	1.29±0.97^c^	1.42±0.57^b^
**Ca (cmol kg** ^**-1**^ **)**	7.89±2.1^b^	7.72±2.1^b^	8.26±2.23^a^	8.11±2.1^a^	7.12±1.9^d^
**Mg (cmol kg** ^**-1**^ **)**	3.52±1.24^d^	3.56±1.4^c^	3.71±1.11^b^	3.62±1.3^c^	3.59±1.12^c^
**Fe (mg kg** ^**-1**^ **)**	0.36±0.12^a^	0.29±0.1^b^	0.26±0.14^b^	0.30±0.03^a^	0.17±0.09^c^
**Mn (mg kg** ^**-1**^ **)**	0.31±0.11^a^	0.27±0.11^b^	0.17±0.03^c^	0.29±0.08^a^	0.24±0.07^b^
**Cu (mg kg** ^**-1**^ **)**	0.16±0.2^a^	0.17±0.01^a^	0.15±0.08^b^	0.16±0.07^a^	0.11±0.1^c^
**Zn (mg kg** ^**-1**^ **)**	0.19±0.11^a^	0.16±0.02^b^	0.14±0.014^c^	0.16±0.04^b^	0.14±0.04^c^

^1^ Mean separation within each row was performed using Duncan’s multiple range test.

^2^ Values followed by different uppercase letters in a row are significantly different (*P* < 0.05) among treatments.

T1, inorganic fertilizer (N:P:K, 2:1:1) + *P*. *aeruginosa* RRALC3; T2, inorganic fertilizer (N:P:K, 2:1:1) + commercial biofertilizer; T3, inorganic fertilizer (N:P:K, 2:1:1); T4, *P*. *aeruginosa* RRALC3; T5, control (no biofertilizer or inorganic fertilizer).

### Nutrient accumulation predicted through logistic equation

Nutrient accumulation in the aerial parts of *P*. *pinnata* seedlings was calculated using the logistic equation. The correlation coefficient (*R*) between the predicted and observed values was very high and confirmed the statistical integrity of the logistic equation. The results of the logistic equation explaining the nutrient accumulation parameters (*V*_m_, *T*_m_, *T*_*a*_, and *T*_*b*_) in the above ground parts of *P*. *pinnata* are shown in Table 4. *T*_*a*_ and *T*_*b*_ are the logistic growth variation points of the two functions reflecting the onset and termination times of nutrient rapid accumulation, respectively. C accumulation in the aboveground biomass showed a rapid change between days 51 and 134 in the T1 treated plants whereas T2 treated plants showed a rapid change between days 62 and 124. From the logistic equation it was deciphered that the rapid C accumulation period of *Pongamia* was 20 days longer because of the application of inorganic fertilizer in combination with *P*. *aeruginosa* (T1) when compared with inorganic fertilizer combined with commercial biofertilizer (T2). The maximum daily C accumulation in seedlings of T1 and T2 treatment was observed on day 98 (4.0 mg plant^−1^ day^−1^) and 77 (3.0 mg plant^−1^ day^−1^), respectively. These results indicated a 14% increase in C accumulation as a result of the T1 treatment.

**Table 4:** Logistic equations and other parameters for nutrient accumulation in the aboveground biomass of *Pongamia pinnata* seedlings.

Nutrient	Treatment	Equation^1^	*V*_m_ ^1^ ^,^ ^2^	*T*_*a*_ ^*1*^ ^,^ ^*2*^	*T*_*b*_ ^*1*^ ^,^ ^*2*^	*T*_m_ ^1^ ^,^ ^2^	*R* ^3^
**Carbon (C)**	**T1**	*W* = 223.345/[1 + e^(5.80−0.0345t)^]	3.956^a^	51^a^	134^a^	98^a^	0.981
	**T2**	*W* = 197.890/[1 + e^(3.46−0.0678t)^]	2.991^b^	62^b^	124^b^	77^b^	0.946
	**T3**	*W* = 162.467/[1 + e^(1.34−0.0123t)^]	2.893^b^	48^c^	82^c^	61^c^	0.945
	**T4**	*W* = 141.623/[1 + e^(2.34−0.0476t)^]	2.471^c^	47^c^	76^d^	54^d^	0.974
	**T5**	*W* = 77.0960/[1 + e^(3.23−0.0910t)^]	1.312^d^	22^d^	61^e^	50^d^	0.937
**Nitrogen (N)**							
	**T1**	*W* = 171.223/[1 + e^(1.80−0.032t)^]	2.123^a^	44^a^	92^a^	76^a^	0.971
	**T2**	*W* = 141.450/[1 + e^(1.83−0.033t)^]	1.816^b^	45^a^	87^b^	69^b^	0.976
	**T3**	*W* = 132.343/[1 + e^(2.31−0.056t)^]	1.623^c^	37^b^	80^c^	51^c^	0.923
	**T4**	*W* = 99.9890/[1 + e^(2.34−0.053t)^]	1.245^d^	24^c^	62^d^	46^d^	0.945
	**T5**	*W* = 87.0930/[1 + e^(3.72−0.027t)^]	0.978^e^	23^c^	62^d^	34^e^	0.978
**Phosphorus (P)**							
	**T1**	*W* = 131.102/[1 + e^(4.80−0.032t)^]	2.160^a^	41^a^	121^a^	76^a^	0.967
	**T2**	*W* = 109.120/[1 + e^(1.23−0.033t)^]	1.977^b^	40^a^	107^b^	71^b^	0.932
	**T3**	*W* = 86.123/[1 + e^(2.31−0.056t)^]	1.672^c^	32^b^	81^c^	62^c^	0.99
	**T4**	*W* = 62.349/[1 + e^(2.34−0.053t)^]	1.345^d^	27^c^	74^d^	52^d^	0.961
	**T5**	*W* = 57.023/[1 + e^(2.12−0.027t)^]	0.912^e^	19^d^	59^e^	47^e^	0.957
**Potassium(K)**							
	**T1**	*W* = 223. 345/[1 + e ^(5.801−0.0345t)^]	1.190^a^	56^a^	103^a^	82^a^	0.987
	**T2**	*W* = 197. 890/[1 + e ^(3.456−0.0678t)^]	1.001^b^	56^a^	97^b^	71^b^	0.965
	**T3**	*W* = 162. 467/[1 + e ^(1.34−0.023t)^]	0.876^c^	43^b^	71^c^	69^b^	0.981
	**T4**	*W* = 141. 623/[1 + e^(2.341−0.0476t)^]	0.671^d^	39^c^	64^d^	54^c^	0.952
	**T5**	*W* = 77.096/[1 + e^(3.230−0.0910t)^]	0.433^e^	37^c^	61^d^	41^d^	0.964

^1^ In the logistic equation, *W* represents the nutrient accumulation of plants (mg/plant), *t* denotes days after the emergence of seedlings, *T*_*a*_ and *T*_*b*_ explain the onset and termination of the rapid nutrient accumulation period, and *V*_m_ denotes the maximum nutrient accumulation (mg plant^−1^ day^−1^) rate.

^2^ Values followed by different uppercase letters in a column are significantly different (*P* < 0.05) among treatments.

Native PGPR, *P*. *aeruginosa* RRALC3. T1, inorganic fertilizer (N:P:K, 2:1:1) + *P*. *aeruginosa* RRALC3; T2, inorganic fertilizer (N:P:K, 2:1:1) + commercial biofertilizer; T3, inorganic fertilizer (N:P:K, 2:1:1); T4, *P*. *aeruginosa* RRALC3; T5, control (no biofertilizer or inorganic fertilizer).

^3^Correlation coefficient (*R*) between the predicted and observed values

N accumulation in the aerial parts of the seedlings was analogous to C accumulation establishing that the T1 treatment had a significant effect. In the logistic equation, the onset time (*T*_*a*_ *)* of N accumulation was different in T1 and T2 at 47 and 45 days, respectively, but the difference was not statistically significant on par with the T2. However, termination time (*T*_*b*_ *)* showed a significant difference: T1 extended the N accumulation period by 7 days and the accumulated amount increased by nearly 7%. During the rapid N accumulation period no significant difference was observed between T4 and T5 but significant differences could be seen in the maximum daily nutrient accumulation (*V*_m_) and maximum daily accumulation time (*T*_m_) in the T4 treated plants.

Despite the dearth of nutrients and inorganic fertilizer, the *P*. *aeruginosa* treated seedlings showed little effect on *T*_*a*_ *T*_*b*_, *V*_m_ and *T*_m_ for P in the above ground biomass of *P*. *pinnata*. The daily P accumulation of T1 and T2 reached a maximum on day 76 (2.160 mg plant^−1^ day^−1^) and day 71 (1.977 mg plant^−1^ day^−1^), respectively. It indicated a P increase of 8.6% due to the T1 treatment.

K accumulation in the aerial parts of *P*. *pinnata* showed rapid changes between day 56 and 103 in T1. The maximum daily accumulation rate of K was 1.190 mg plant^−1^ day^−1^ in T1 as deduced from the logistic equation. T_a_ and T_b_ were statistically significant in T4 and T5; however V_m_ and T_m_ were statistically on a par with each other. In logistic equation accumulation of C, N, P and K concentration and the growth parameters (*V*_m_, *T*_m_, *T*_*a*_, and *T*_*b*_) of *P*. *pinnata* were statistically different in T3 (Inorganic fertilizer alone) and T4 (*P*. *aeruginosa* alone) treatments.

## Discussion

Currently, the major threat to replantation in forests is topsoil loss due to erosion. This creates a situation in which there is a decrease in the number of beneficial plant-associated microorganisms present to sustain plant growth [44]. Heavy or above-average rainfall alone is not enough to promote good plant growth and to enhance the microbial population even in the case of desert soils [18]. Thus, the planned reintroduction of native PGPB would definitely be an ideal solution for plant and soil nutrient enhancement in degraded forests.

Plant rhizosphere and PGPB interactions are essential to enhance plant growth and soil fertility and diverse ranges of PGPB stimulate growth through various direct and indirect mechanisms [45]. Several studies have reported that PGPB is effective in promoting plant growth and enhancing the nutrient content and yield of agricultural crops [46, 47]. To date, very few studies have been reported on the interaction between forest seedlings and PGPBs [48, 49]. However, there has been some research conducted on the application of PGPB in degraded forest soils and forest seedlings in nursery tree plants [50]. In the present study, a PGP bacterium *P*. *aeruginosa* RRALC3 was isolated from the NRF. Indigenous PGPB are well adapted to native or local conditions though no commercial PGPB products are known to be effective on desert soils [51, 52] and the addition of non-indigenous microbes to the soil has the potential to impact the indigenous rhizosphere population [53]. Due to their excellent growth promotion and biocontrol activities *P*. *aeruginosa* isolated from the rhizosphere of crop plants and environmental samples can be used as inoculant for promotion of plant growth [54–56]. *P*. *aeruginosa* is frequently found in soils [57] and is known to colonize cucumber roots [58], lettuce leaves [57], and the roots of sweet basil [59], sugar beet [60], wheat [58] and Arabidopsis [60].

Plants, especially their endospheres and rhizospheres are important reservoirs for emerging opportunistic pathogens [61,62]. The majority of rhizobacterial species which emerged as pathogens belongs to the group of antagonistic bacteria, e.g., *Burkholderia*, *Enterobacter*, *Pseudomonas*, *Ralstonia*, *Serratia*, *Staphylococcus* and *Stenotrophomonas* that enter bivalent interactions with plant and human hosts. Several members of these genera show plant growth promoting as well as excellent antagonistic properties against plant pathogens; therefore, they are utilized to control pathogens to promote plant growth [61]. Many mechanisms involved in the interaction between antagonistic plant associated bacteria and their host plants are similar to those responsible for pathogenicity of bacteria [63]. *P*. *aeruginosa* is more widely known as an opportunistic pathogen for humans and animals than as a soil bacteria. In this regard, *P*. *aeruginosa* can produce severe infections in immunocompromised hosts [64] and is the major factor for morbidity and mortality in cystic fibrosis patients [65].

Opportunistic pathogens occur in natural environments and are often associated with other eukaryotic hosts such as plants. They are often characterized by several of the following properties: (i) r-strategists = copiotrophs, (ii) cultivable, (iii) antagonistic towards other microorganisms, (iv) highly competitive, (v) highly versatile in their nutrition, (vi) hypermutators, (vii) resistant against antibiotics and toxins and (viii) form biofilms [66]. Similarly, *P*. *aeruginosa* RRALC3 isolated from this study also possessed the multiple plant growth promoting traits such as nitrogen fixation, IAA production and phosphate solubilization. Blood agar is widely used for the isolation of bacteria from clinical sample. Definitive clinical *P*. *aeruginosa* isolates could exhibit heamolytic activity in blood agar [67]. In this study, *P*. *aeruginosa* RRALC3 didn’t show heamolytic activity in blood agar. Generally highest percentage of heamolytic activity increased the pathogenicity of the *P*. *aeruginosa* [68]. Previously, it was reported that *P*. *aeruginosa* strains producing clear haemolysis within the first 48 h of incubation. It should be noted that not all clinical *P*. *aeruginosa* isolates are haemolytic; the percentage of environmental strains being haemolytic was similar to that reported for clinical *P*. *aeruginosa* isolates [69,70]. Most of the clinical *P*. *aeruginosa* strains produced proteases and could invade epithelial cells in a cell culture model system. These strains contained multidrug resistance determinants, and presented genes from the quorum-sensing and type III secretion systems. Some of them expressed either haemolytic or proteolytic activities or both. These characteristics are typical of virulent strains; however, they were also present in the environmental strains [62]. *P*. *aeruginosa* produces a variety of extracellular enzymes contributing to its pathogenicity. Elastase is an enzyme capable of degrading or inactivating important biological tissues and immune system components, including immunoglobulin. It also helps the survival of *P*. *aeruginosa* in various settings. *ybtQ* gene is present only in pathogenic strains of *P*. *aeruginosa*. The *ecfX* gene encodes an ECF (extracytoplasmic function) sigma factor suitable for species-specific identification of pathogenic *P*. *aeruginosa* isolates as it can act as a virulence factor in clinical isolates [32–34]. The *P*. *aeruginosa* isolate RRALC3 used as a PGPB in this study did not possess pathogenic genes to cause disease, and was phylogenetically clustered with other environmental isolates. Nematode *Caenorhabditis elegans* is a highly valuable model for the study of bacterial pathogenecity [71,72]. Initially this model was developed to identify genes in *P*. *aeruginosa* that are important for pathogenesis [73]. Upon inoculation of pathogenic bacterial strain on *C*. *elegans* which should show sick appearance at day 2 including reduced movement capacity; distended intestine. Percentage of live worms after 2 days ≤50%. Total number of dead aworms after 5 days ≤100%. A strain is considered pathogenic when at least one of the above criteria can be observed [74]. The *C*. *elegans* assay can be integrated into initial screenings for biological control agents and is a new tool to identify effects against eukaryotes in a very early stage of the product development [74]. Although could seems irrational to study lung diseases in animals (*C*. *elegans* and Zebrafish [*Danio rerio*]) who do not have lungs, several evidences support the benefits that these studies, if carried properly, may to bring in the elucidation of human lung pathology diseases [75]. In future, before development of biological control agent and or bio-inoculant with isolated *P*. *aeruginosa* RRALC3 should be tested in a suitable animal model for its virulence.

The application of *P*. *aeruginosa* strains AW8 and LS3 is known to enhance the shoot, root, and panicle growth as well as plant height in the case of wheat plants [76,77]. In cowpea, *P*. *aeruginosa* FP6 leads to increased germination of seeds and plant shoot and root [78]. *P*. *aeruginosa* strain PS1 inoculation has led to enhanced growth parameters of green gram in insecticide stressed soils [79]. In okra, tomato and African spinach, *P*. *aeruginosa* stimulated the availability nutrients and enhanced the composition of root exudates [80].

As hypothesized by Bashan *et al*. [18], native leguminous trees are essential for the replantation of highly eroded, degraded lands and they respond favorably to inoculation with PGPB in a manner similar to their application in agriculture and agroforestry. In this study, the leguminous tree *P*. *pinnata* was used as a test plant. It was observed that the promotion of shoot and root growth in *Pongamia* seedlings was greater under the cumulative effects of chemical fertilizer and native PGPB as compared to inorganic fertilizer and commercial biofertilizer treatments. Similar results were obtained by Vanessa *et al*. [81] in wheat plants using indigenous Actinobacteria microflora and commercial products. PGPB acts directly on plant roots through the production of indole acetic acid. This phytohormone is supposed to stimulate the formation of lateral roots and increase nutrient absorption from root hairs [61]. Generally, IAA is a plant growth hormone produced by microbes; it is non-essential for microbial function and growth but maximizes plant—microbe interactions [82]. In this study, the native bacterial strain produced IAA and exhibited enormous potential for promoting growth in the above and below ground biomass of plants.

Chemical fertilization of seedlings with commercial biofertilizer resulted in a significant increase in biomass when compared with the control but was shown to be less effective than the combined application of *P*. *aeruginosa* RRALC3 and chemical fertilizer. Without fertilizer, the native PGPB showed a statistically significant difference at the *P* < 0.05 level. This difference was higher compared to the control and lower compared to seedlings treated with commercial biofertilizer. When water alone was applied to unfertilized seedlings treated with PGPB, the nutrient level of the soil was not optimal for plant growth. A similar result was also reported by Liu et al. [19] in *Fraxinus americana* container seedlings treated with *Bacillus spp*. without chemical fertilizer.

In this study, treatment with chemical fertilizer and a native PGPB was found to increase the biomass and major nutrient content of *P*. *pinnata* seedlings. PGPB have the potential to enhance water and nutrient absorption from the soil which in turn increases shoot and root growth in plants [83,84]. Different researchers [85,86] have also observed a similar positive effect on the yield and growth of sweet cherry and apple trees when treated with *Bacillus* OSU-142 and BA-8, respectively.

Bashan *et al*. [18] reported the revegetation of desert soil using three native leguminous trees (*Prosopis articulata*, *Parkinsonia microphylla*, and *Parkinsonia florida*) together with two PGPB (*Azospirillum brasilense* and *Bacillus pumilus*) and an arbuscular mycorrhizal fungus when supplemented with limited amounts of compost and water. Their results showed that plant survival and growth were improved under desert soil conditions using native PGPB. The increase in total biomass and the plant growth enhancement effects of the native bacteria used in this study on the legume *P*. *pinnata* could be explained by the presence of multiple plant growth promoting traits. In addition, in the present study, it was found that inoculation with *P*. *aeruginosa* RRALC3 increased the P, Mg, S, Ca and Zn content of *P*. *pinnata* seedlings. This might be attributed to the production of organic acids by the plants themselves as well as by various bacteria in the rhizosphere as production of organic acids is instrumental in decreasing the soil pH and stimulating the availability of micronutrients as previously supported by findings from other research groups [87,88] as well.

Plant root exudates are powerful microbial growth signals and crucial because of their impact on nutrient acquisition by plants [89]. PGPB produces IAA which causes plant cell walls to loosen resulting in an increased amount of root exudation that provides additional nutrients to support the growth of plants and rhizosphere bacteria [90]. Root exudates contain various chemical molecules that mobilize the availability of P and Fe in nutrient deficient soils [89]. In the present study, chemical fertilization in combination with PGPB enhanced the production of root exudates which probably led to the substantial increase in plant nutrient and carbon contents in *P*. *pinnata*.

In summary, soil treated with the native PGP bacterium *P*. *aeruginosa* RRALC3 in combination with inorganic fertilizer enhanced the growth, nutrient and carbon content of the native legume *P*. *pinnata*. The native PGPB along with inorganic fertilizer was the most effective treatment in the NRF indigenous soil. In nutrient deficient conditions, administration of native PGPB alone had less but still substantial effect when compared to the control. Hence, this study emphasizes on the need to employ native beneficial microorganisms in combination with adequate inorganic fertilizer in the replantation of degraded forests.

## Supporting Information

S1 Fig Ammonia production by Diazotrophs isolated from degraded forest soils using the various N free semi solid media a) LGI b) JMV, c) Nfb. (TIF)

S2 Fig Detection of nif H gene in *Pseudomonas aeruginosa* RRALC3 by PCR with Nif gene: lane 1: 1kb ladder, lane 2: negative control (DH5α), lane 3: *Pseudomonas aeruginosa* (RRALC3), lane 4: *Azospirillum brasilens* MTCC 125 (Positive control). (TIF)

S3 Fig Pathogenecity assay for examination of virulence genes in (a) *ecfX* (b) *ybtQ* and (c) *lasB*. Lane 1: 1 kb ladder, lane 2: negative control (DH5α), lane 3: pathogenic *Pseudomonas aeruginosa* PA14, lane 4: *P*. *aeruginosa* RRALC3, lane 5: non-pathogenic *P*. *aeruginosa* PA01.
(TIF)

S4 Fig Hemolytic activity of *Pseudomonas aeruginosa* isolates (PA01, PA14, RRALC3) in blood agar plates a) *Pseudomonas aeruginosa* PA01- no hemolytic (ɤ) activity, b) *Pseudomonas aeruginosa* PA14 –dark greenish colour α hemolytic activity, c) *Pseudomonas aeruginosa* RRALC3- no hemolytic (ɤ) activity. (TIF)
