# Supplementary figures and images for: Pseudomonas aeruginosa RRALC3 Enhances the Biomass, Nutrient and Carbon Contents of Pongamia pinnata Seedlings in Degraded Forest Soil
Source: PLoS One. 2015 Oct 13;10(10):e0139881. doi: 10.1371/journal.pone.0139881 (PMC4604145; doi:10.1371/journal.pone.0139881)

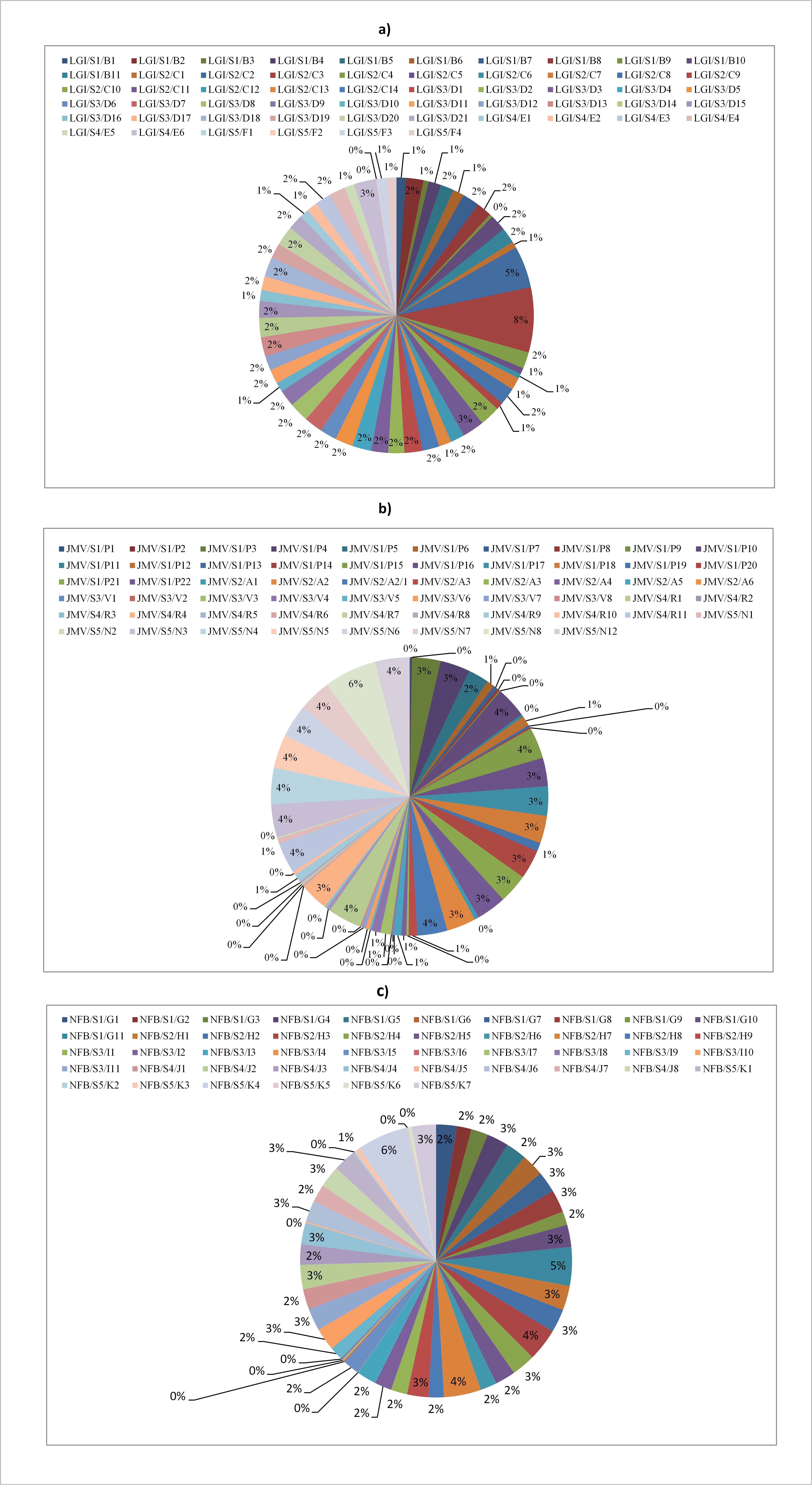

Supplement: S1 Fig — (TIF) [file pone.0139881.s001.tif]

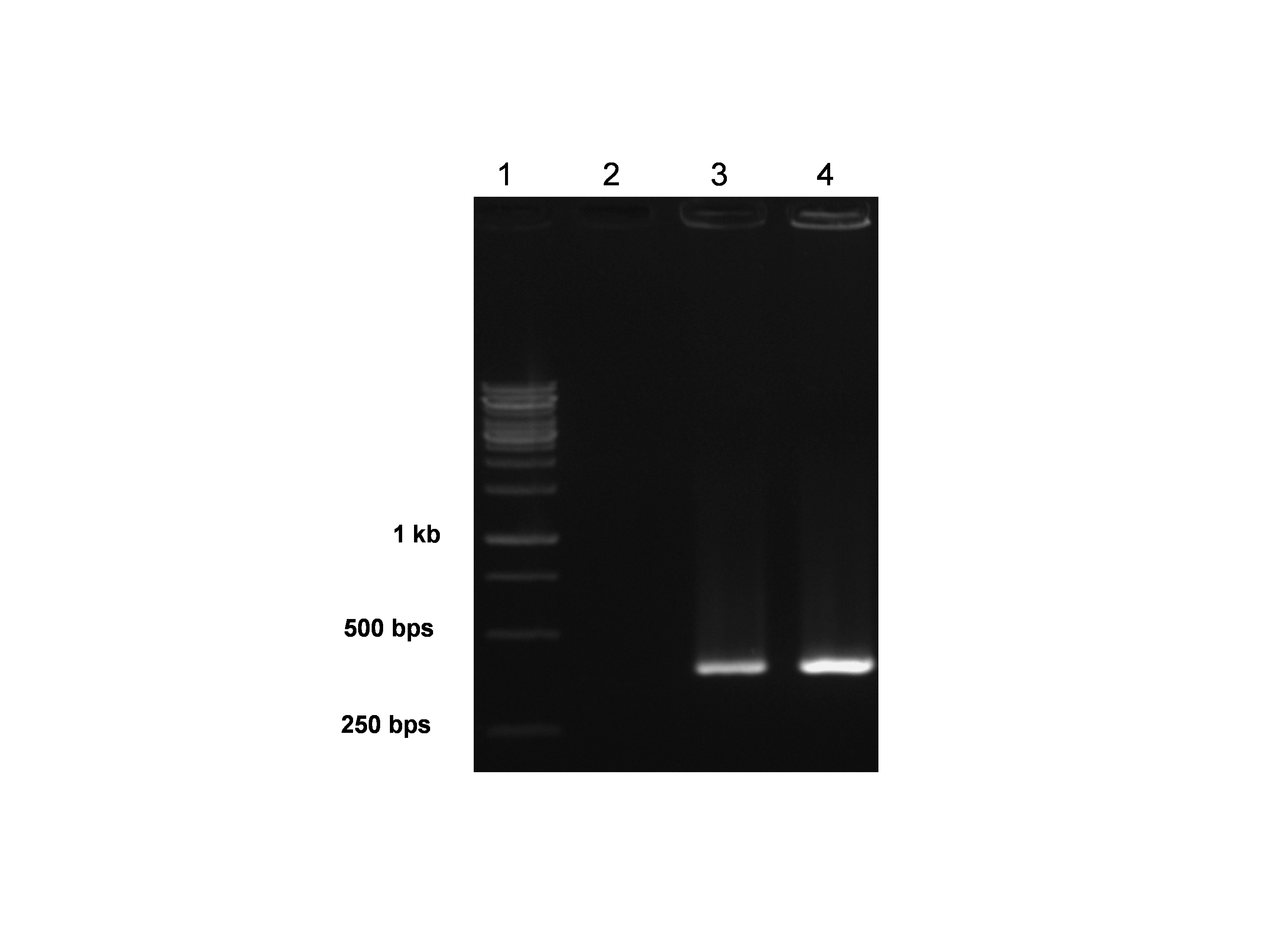

Supplement: S2 Fig — (TIF) [file pone.0139881.s002.tif]

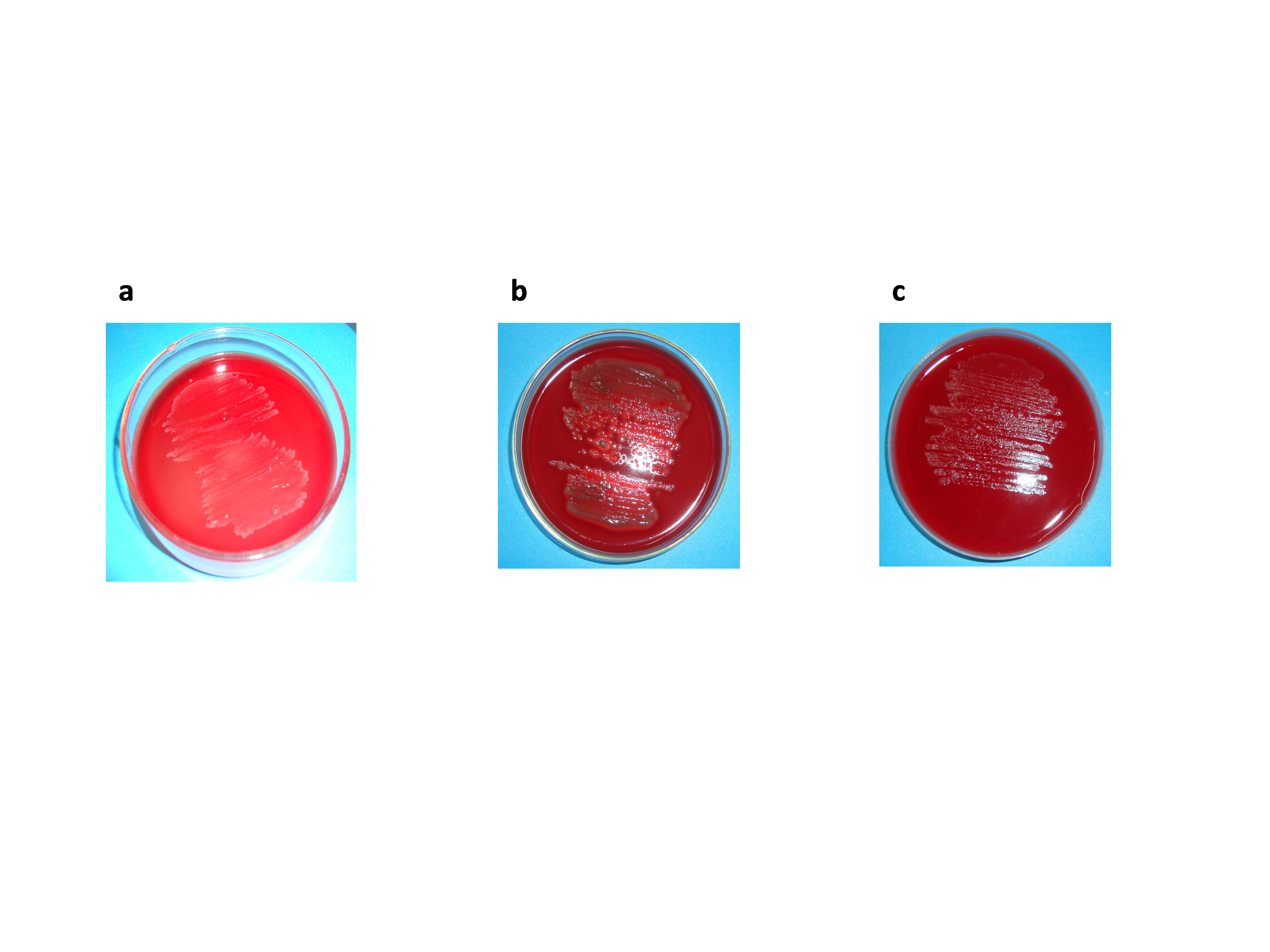

Supplement: S3 Fig — Lane 1: 1 kb ladder, lane 2: negative control (DH5α), lane 3: pathogenic Pseudomonas aeruginosa PA14, lane 4: P. aeruginosa RRALC3, lane 5: non-pathogenic P. aeruginosa PA01. (TIF) [file pone.0139881.s003.tif]

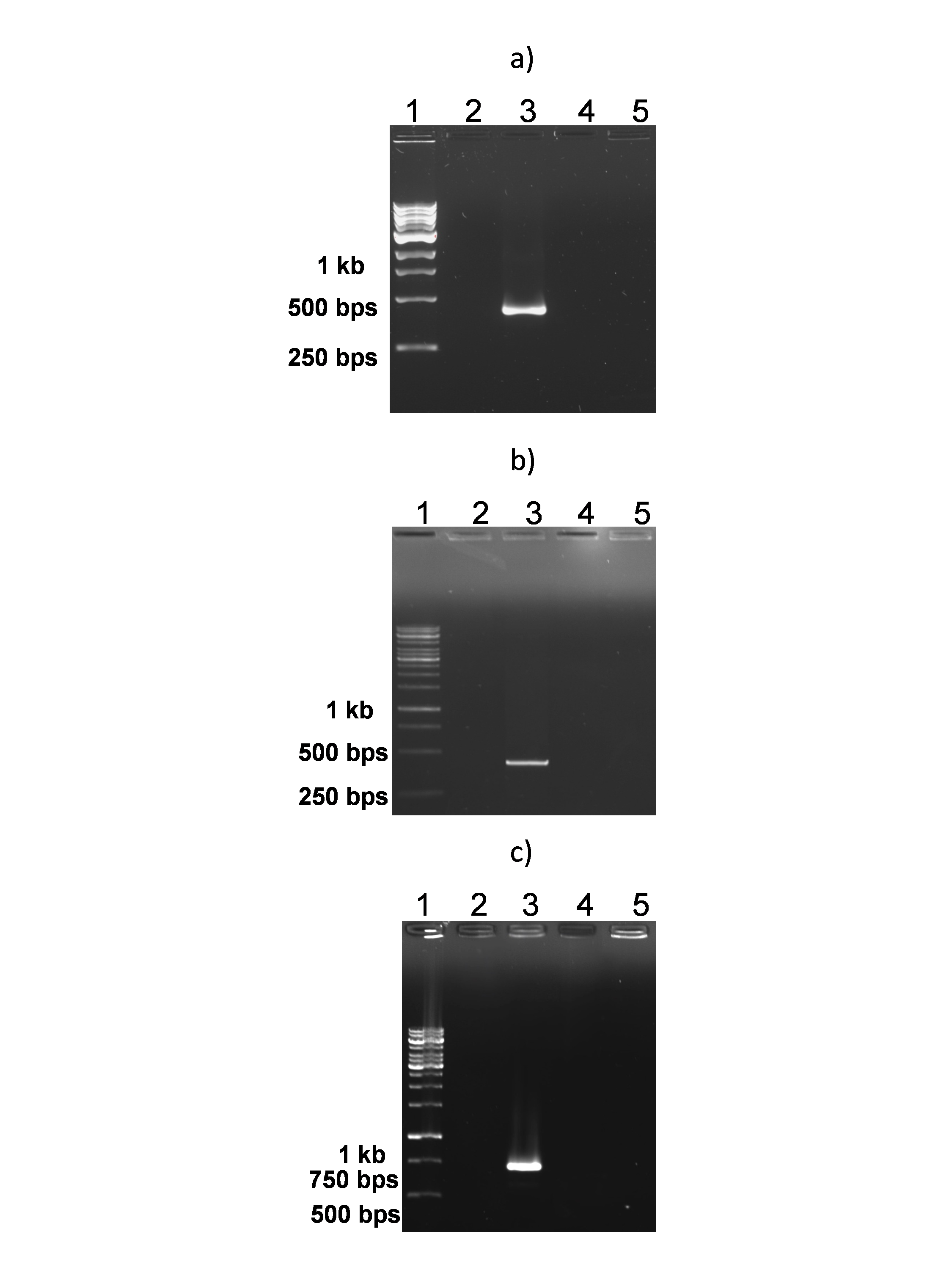

Supplement: S4 Fig — (TIF) [file pone.0139881.s004.tif]
